# Response of Medical Cannabis (*Cannabis sativa* L.) to Nitrogen Supply Under Long Photoperiod

**DOI:** 10.3389/fpls.2020.572293

**Published:** 2020-11-17

**Authors:** Avia Saloner, Nirit Bernstein

**Affiliations:** ^1^Institute of Soil, Water and Environmental Sciences, Volcani Center, Rishon LeZion, Israel; ^2^Faculty of Agriculture, Food and Environment, The Hebrew University of Jerusalem, Rehovot, Israel

**Keywords:** cannabis, fertilizer, nutrition, nitrogen, nitrate, nitrogen use efficiency, photoperiod, vegetative

## Abstract

The development progression of medical cannabis plants includes a vegetative growth phase under long photoperiod, followed by a reproductive phase under short photoperiod. Establishment of plant architecture at the vegetative phase affects its reproduction potential under short photoperiod. Nitrogen (N) is a main component of many metabolites that are involved in central processes in plants, and is therefore a major factor governing plant development and structure. We lack information about the influence of N nutrition on medical cannabis functional-physiology and development, and plant N requirements are yet unknown. The present study therefore investigated the developmental, physiological, and chemical responses of medical cannabis plants to N supply (30, 80, 160, 240, and 320 mgL^−1^ N) under long photoperiod. The plants were cultivated in an environmentally controlled growing room, in pots filled with soilless media. We report that the morpho-physiological function under long photoperiod in medical cannabis is optimal at 160 mgL^−1^ N supply, and significantly lower under 30 mgL^−1^ N, with visual deficiency symptoms, and 75 and 25% reduction in plant biomass and photosynthesis rate, respectively. Nitrogen use efficiency (NUE) decreased with increasing N supply, while osmotic potential, water use efficiency, photosynthetic pigments, and total N and N-NO_3_ concentrations in plant tissues increased with N supply. The plant ionome was considerably affected by N supply. Concentrations of K, P, Ca, Mg, and Fe in the plant were highest under the optimal N level of 160 mgL^−1^ N, with differences between organs in the extent of nutrient accumulation. The majority of the nutrients tested, including P, Zn, Mn, Fe, and Cu, tended to accumulate in the roots > leaves > stem, while K and Na tended to accumulate in the stem > leaves > roots, and total N, Ca, and Mg accumulated in leaves > roots > stem. Taken together, the results demonstrate that the optimal N level for plant development and function at the vegetative growth phase is 160 mgL^−1^ N. Growth retardation under lower N supply (30–80 mgL^−1^) results from restricted availability of photosynthetic pigments, carbon fixation, and impaired water relations. Excess uptake of N under supply higher than 160 mgL^−1^ N, promoted physiological and developmental restrictions, by ion-specific toxicity or indirect induced restrictions of carbon fixation and energy availability.

## Introduction

The *Cannabis sativa* industry is rapidly evolving worldwide, with an escalating demand for agricultural products for the medicinal and recreational markets (UNODC, 2018; Chouvy, 2019). In spite of the long-time use by humanity for medical, commercial, and recreational purposes, science-based information on the cannabis plant is restricted due to its legal state. Understanding of the plant biology and physiology is needed for supporting the development of modern cultivation schemes.

Phenological stages of cannabis development have been proposed by several authors (Farag and Kayser, 2017; Mishchenko et al., 2017; Raman et al., 2017; Spitzer-Rimon et al., 2019). The development progression includes establishment of the vegetative plant body under long photoperiod, followed by a reproductive development stage under short photoperiod. During growth under long photoperiod, the main stem of the cannabis plants branches monopodially, producing alternate lateral branches. The lateral branches produce second- and third-order branches. Following a transition to short photoperiod, the development of the shoot changes, and the apical buds of the main stem and of primary branches, as well as lateral buds, develop inflorescences that are the basis for commercial yield in drug-type cannabis. Plant architecture at the end of the long photoperiod therefore profoundly affects its reproduction potential and hence yield capacity under short photoperiod. In horticultural practice, cannabis is propagated by rooted cuttings (Cervantes, 2006; Caplan, 2018) and many nurseries are therefore focusing nowadays on growth under long photoperiod, in an attempt to produce high-standard plants.

Mineral nutrition is one of the main factors affecting vegetative development of the plant body (Lea and Morot-Gaudry, 2001; Hawkesford et al., 2012), which dictates the reproductive potential. Understanding the nutritional needs and the physiological and morphological responses of the medical cultivars to mineral nutrition will aid in improving schemes for nutrient supply and plant function. Nitrogen (N) is the most abundant mineral element in plants (Ohyama, 2010). Due to its many roles in plant metabolism, including protein, chlorophyll, and nucleic acid synthesis, N supply is one of the key factors regulating plant growth (Hawkesford et al., 2012). As a result, plant development and metabolism are highly affected by N supply, and N nutrition is one of the prime environmental factors influencing plant function and production (Hauck et al., 1984; Lea and Morot-Gaudry, 2001). Therefore, we chose to examine the response of medical cannabis to N supply in the vegetative growth phase.

Very little information is available about N nutrition of medical cannabis. Available studies into N effects on *C. sativa* focused mainly on hemp, cultivated for fiber or seed production. Optimal N inputs for fiber growth were reported by numerous studies to range from 60 to 150 kg N ha^−1^, and higher N levels to increase fiber yield but impair fiber strength (Ehrensing, 1998). Plant biomass, seed yield, and protein contents were reported to increase with N supply up to 120 kg N ha^−1^ (Vera et al., 2004) or 200 kg N ha^−1^ (Aubin et al., 2015), and plant growth and inflorescence size increased up to 240 kg N ha^−1^ supply (Papastylianou et al., 2018). Contradictory results were reported for N effects on secondary metabolism in hemp: N supply (between 0 and 125 ppm) did not affect THC contents in inflorescences (Coffman and Gentner, 1977), while high N supply [above 450 mg N kg(soil)^−1^] reduced THC contents in leaves (Bócsa et al., 1997). Moreover, N supply was reported to effect sex expression in hemp (Van der Werf and Van den Berg, 1995). It should be considered that hemp has been domesticated and bred for fiber production and hence has different genetic, morphological, and developmental characteristics than the drug-type cannabis that likely entail different nutritional needs. Therefore, it is difficult to derive the drug-type response from information obtained for hemp.

Only a few studies investigated the impact of mineral nutrition in general, and N nutrition in particular, on drug-type cannabis. Recent studies from our group shed some light on various aspects of mineral nutrition influence on medical cannabis development, physiology, and secondary metabolism. We demonstrated an interplay between plant morphology, physiology, and chemistry throughout the plant (Bernstein et al., 2019a), and reported variability along the plant height and between plant organs in cannabinoid contents (including THC and CBD), concentrations of inorganic nutrients, and physiological characteristics (Bernstein et al., 2019a). We showed, for the first time, that nutritional supplements such as humic acids, NPK, and P induce changes in cannabinoid contents in medical cannabis (Bernstein et al., 2019b). In a study that focused on K nutrition, we reported that the response of medical cannabis to K at the vegetative growth phase varied between genotypes, revealing genetic differences within the *C. sativa* species to mineral nutrition (Saloner et al., 2019); 60 mgL^−1^ K was the lowest K concentration optimal for growth and metabolism; and 240 ppm was excessive and detrimental for development and function in one genotype, but stimulated rather than restricted development in the other genotype (Saloner et al., 2019). Additionally, under organic fertilization, 389 mgL^−1^ was the optimal level of N for growth and yield production (Caplan et al., 2017). The extent of N accumulation in the plant was higher in the leaves > roots > stem (Saloner et al., 2019).

The present study focused on N nutrition at the vegetative growth stage. The hypothesis guiding the workplan was that N supply induces morphological and developmental changes in medical cannabis that are associated with changes in the plant ionome, carbon fixation, and tissue water relations. To test this hypothesis, we investigated effects of N inputs ranging from 30 to 320 mgL^−1^ N in the nutrient solution on physiological and morphological characteristics and mineral accumulation in the plant organs. The information obtained contributes to our understanding of medical-cannabis physiology and mineral nutrition, and directs optimal fertigation regimes for improved production.

## Materials and Methods

### Plant Material and Growing Conditions

The medical cannabis (*C. sativa* L.) cultivar “Annapurna” (Canndoc Ltd., Israel), which is approved for commercial medical use in Israel, was used as a model system in this study. It contains similar concentrations of THC and CBD (about 7%) and demonstrates predominantly indica characteristics. Plants were propagated from cuttings of a single mother plant in coconut fiber plugs (Jiffy international AS, Kristiansand, Norway); 24 days thereafter, the rooted cuttings were planted in 3 L plastic pots, in perlite 2-1-2 cultivation media (Agrekal, Habonim, Israel) and the fertilization treatments were initiated 1 day thereafter. The plants were then divided into five treatments of N supply: 30, 80, 160, 240, and 320 ppm N, five plants per treatment, and grown for an additional 32 days under 18/6 h light/dark photoperiod in a controlled environment growing room. The size of the plant at the end of the vegetative phase has a direct impact on morpho-development at the reproductive phase which impact standardization of secondary metabolites in the plant. To increase standardization, the vast majority of the medical cannabis growers regulate the mature plant size, by keeping the vegetative period short, usually between 14 and 21 days. The duration of the study, 32 days, was selected to include the conventional cultivation period for the vegetative growth period. Light was supplied by Metal Halide bulbs (400 μmol^∗^m^–2*^s^−1^, Solis Tek Inc, Carson, CA, United States). Temperatures in the cultivation room were 28 and 25°C day/night, respectively, and the relative humidities were 42 and 49%, respectively. The volume of irrigation in each irrigation was 330–650 mL/pot, set to allow 30% of drainage (leachate). The plants were irrigated daily. Irrigation was supplied via a 1 L h^−1^ discharge-regulated drippers (Netafim, Tel-aviv, Israel), one dripper per pot. Fertilizers were supplied by fertigation, i.e., dissolved in the irrigation solution at each irrigation. To ensure high accuracy of mineral concentrations at the various treatments, the fertigation solutions were hand-prepared and the irrigation (fertigation) was thus conducted from final solutions. The irrigation was conducted with an open cycle. The irrigation solution contained (in mM): 5.1 K^+^, 1.9 P-PO_4_^−2^, 1.12 Ca^+ 2^, 1.83 Mg^+ 2^, 3.25 Na^+^, 2.77 S-SO_4_^−2^, 2.57 Cl^−^, 0.03 Fe^+ 2^, 0.014 Mn^+ 2^, 0.006 Zn^+ 2^, 0.0006 Cu^+ 2^, 0.0003 Mo^+ 2^, and 0.009 B^+ 3^. N was supplied in the concentrations of 2.1, 5.7, 11.4, 17.1, and 22.9 mM [e.g., 30, 80, 160, 240, and 320 mgL^−1^ (ppm), respectively], 80% in the form of N-NO_3_^–^ and 20% as N-NH_4_^+^. Iron was supplied chelated with EDDHSA, Zn, Mn, and Cu were chelated with EDTA, and B and Mo were added with the fertilizers B-7000 and Bar-Koret (Israel chemicals, Tel-Aviv, Israel), respectively.

The experiment was arranged following a completely randomized design. All measurements were conducted with five replicates following the experimental design and results are presented as average ± standard error (SE).

### Inorganic Mineral Analysis

Inorganic mineral concentrations in the plant organs were analyzed by destructive sampling 32 days after the initiation of the N fertigation treatments. The dissected shoots were rinsed twice with distilled water and blotted dry and leaves were separated from the stems. Roots were separated from the growing media, gently rinsed three times in distilled water and blotted dry. Fresh and dry biomass was measured with a Precisa 40SM-200A balance (Zurich, Switzerland). Dry weights were determined following desiccation at 64°C for 48 h, and the dry tissue was ground to a powder.

The plant samples were analyzed for concentrations of N, N-NO_3_, P, K, Ca, Mg, Fe, Mn, Zn, Cu, and Na. Three different procedures were used for extraction of the inorganic mineral elements from the ground plant tissue. For the analysis of Ca, Mg, Fe, Zn, Cu, and Mn, the ground plant tissue was digested with HClO_4_ (70%), and HNO_3_ (65%), and the elements were analyzed with an atomic absorption-emission spectrophotometer (AAnalyst 400 AA Spectrometer, PerkinElmer, Massachusetts, United States). For the analysis of N, P, K, and Na, the dry ground tissue was digested with H_2_SO_4_ (98%) and H_2_O_2_ (70–72%). Na and K were analyzed by a Flame Photometer (410 Flame Photometer Range, Sherwood Scientific Ltd., The Paddocks, United Kingdom), and N and P by an Autoanalyzer (Lachat Instruments, Milwaukee, WI, United States). For the analyses of N-NO_3_, 0.1 g dried plant samples were extracted with 50 mL of distilled water, then reacted with an N-NO_3_-specific reagent Szechrome NAS (Polysciences Inc., Eppelheim, Germany) to create a color reaction, and absorbance at 570 nm was measured by a 10 UV Scanning spectrophotometer (Genesys, Thermo scientific, Waltham, MA, United States).

Routine monitoring of mineral concentrations, electrical conductivity (EC), and pH in the irrigation solutions and the leachates were conducted throughout the experiment. Mineral concentrations in irrigation and drainage solutions were performed as described for the plant extraction and digestion solutions.

### Physiological Parameters

The plants were sampled for physiological analyses 32 days after the initiation of the fertigation treatments.

#### Determination of Osmotic Potential

The youngest mature fan leaf on the main stem located at the fourth node from the top of the stem was carefully removed, washed twice in distilled water, and blotted dry. The two most peripheral leaflets were dissected from the leaf and placed into a 1.7 mL micro-test-tube. The tubes were frozen in liquid nitrogen and stored at −20°C. For the analyses, the frozen tissue was crushed inside the tubes, the bottom of the tubes was pin-pricked, and the tubes, set inside another 1.7 mL tube, centrifuged for 5 min in a refrigerated centrifuge (Sigma Laboratory Centrifuges, Germany) at 4°C at 6000 r/min. Fifty microliters of the fluids collected in the lower tube were used for osmotic potential measurement with a cryoscopic microosmometer (Osmomat 3000, Gonotec, Berlin, Germany). Five replicated leaves from five replicated plants per treatment were analyzed.

#### Determination of Membrane Leakage

Ion leakage from the leaf tissue as an indicator of stress-induced membrane injury (Lu et al., 2008) was measured following Shoresh et al. (2011) with minor modifications. The youngest mature fan leaf on the main stem of the plant, from the fourth node from the top of the plant, was washed twice in distilled water and blotted dry. The middle leaflet was placed in a 50 mL plastic tube containing 30 mL of distilled water and shaken for 24 h. The EC of the solution containing the leaf was measured by a conductivity meter (Cyberscan CON 1500, Eutech Instruments Europe B.V., Nijkerk, Netherlands). The samples were then autoclaved for 30 min, allowed to cool down at room temperature for 45 min, shook for an additional 1 h, and the EC of the solution was measured again. Ion leakage from the plant tissue was calculated as percentage (%) of the post autoclave EC value from the initial pre-autoclave value. Presented results are averages of five replicated plants per treatment.

#### Analysis of Photosynthetic Pigments

The youngest mature fan leaf on the main stem located at the fourth node from the top of the stem was used for chlorophyll *a* and *b* and carotenoids analysis. The leaf was washed twice in distilled water and blotted dry. Five discs, each 0.6 cm in diameter, were cut from the second-largest leaflet, placed in 0.8 mL 80% (v/v) ethanol, and kept in −20°C until further analysis. For the analysis, the samples were heated for 30 min at 90°C and the extract was collected in 2 mL micro test-tubes. The remaining tissue was extracted again in 0.5 mL 80% (v/v) ethanol at 100°C for 30 min, and the combined extract was mixed by vortex. Next, 0.4 mL of extract was transferred to 5 mL 80% (v/v) acetone, and absorbance at 663, 646, and 470 nm was measured by a 10 UV Scanning spectrophotometer (Genesys, Thermo scientific, Waltham, MA, United States). Chlorophyll *a* and *b* and carotenoids concentrations were calculated following Lichtenthaler and Wellburn (1983).

#### Determination of Relative Water Content

The second youngest mature fan leaf on the main stem, located at the fifth node from the top of the stem, was used for the analysis of relative water content (RWC). The leaf was carefully removed from the plant and immediately weighed (Precisa 40SM-200A balance, Zurich, Switzerland). The leaf was then placed in a 50 mL plastic tube filled with distilled deionized water. The tubes were kept at room temperature for 24 h and then the leaves were blotted-dry and weighed again. Dry weights were obtained following desiccation for 48 h at 64°C. RWC was calculated following Bernstein et al. (2010) as of Eq. 1. The analyses were conducted for five replicated plants per treatment.

(1) $$\begin{array}{c} RWC=\left(FW-DW\right)/\left(TW-DW\right) \end{array}$$

where RWC is relative water content, FW is fresh weight, DW is dry weight, and TW is turgid weight.

#### Plant Architecture and Development

Stem diameter, plant height, and the number of nodes on the main stem were measured five times throughout the experiment; 0, 7, 14, 20, and 30 days following the initiation of the fertilization treatments. Side branches length was measured 14, 20, and 30 days after the initiation of the fertigation treatments. Branch length and plant height were measured from the base of the plant to the top of the branch with a ruler. Stem diameter was measured with a digital electronic caliper (YT-7201, Signet Tool International Co., Ltd., Shengang District, Taiwan) at the location 3 cm from the plant base. For side branches length, the four lowest branches on the main stem were measured from their point of emergence at the main stem, and their length was averaged to give the plant value. The measurements were conducted on five replicated plants, following the experimental design.

#### Photosynthesis and Transpiration, Intrinsic Water Use Efficiency, Stomatal Conductance, and Intercellular CO_2_ Concentration

Net photosynthesis rate, transpiration rate, stomatal conductance, and intercellular CO_2_ concentration were measured on the youngest mature fan leaf on the main stem, from the fourth node down the plant’s top. The measurements were conducted with a Licor 6400 XT system (LI-COR, Lincoln, NE, United States), 23 days after the initiation of the fertilization treatments. The photosynthesis and stomatal conductance results were used for the calculations of intrinsic water use efficiency (WUEi), which is the response of WUE at the leaf level. It was calculated as net photosynthetic rate divided by stomatal conductance. The measurements were conducted on five replicated plants.

### Plant Fresh and Dry Biomass

Biomass accumulation in the vegetative shoot organs, i.e., leaves, stems, and roots, was evaluated by destructive sampling three times throughout the experiment; 0, 16, and 32 days following the initiation of the fertigation treatments. Dry weights were measured following desiccation in 64°C for 48 h. Nitrogen use efficiency (NUE) was calculated as the total dry weight of the plant on day 32 divided by the cumulative amount of N (g/plant) supplied to the plant throughout the experiment duration. Presented results are averages ± SE for five replicated plants.

### Statistical Analyses

The data were subjected to one-way or two-way analysis of variance (ANOVA) followed by Tukey’s HSD test. A comparison of relevant means was conducted by Fisher’s least significant difference (LSD) test at 5% level of significance. In figures presenting two-way ANOVA results, the bars represent the LSD between means at *P* ≤ 0.05. The analysis was performed with the Jump software [Jump package, version 9 (SAS 2015, Cary, NC, United States)].

## Results

### Plant Visual Characteristics

**FIGURE 1:**
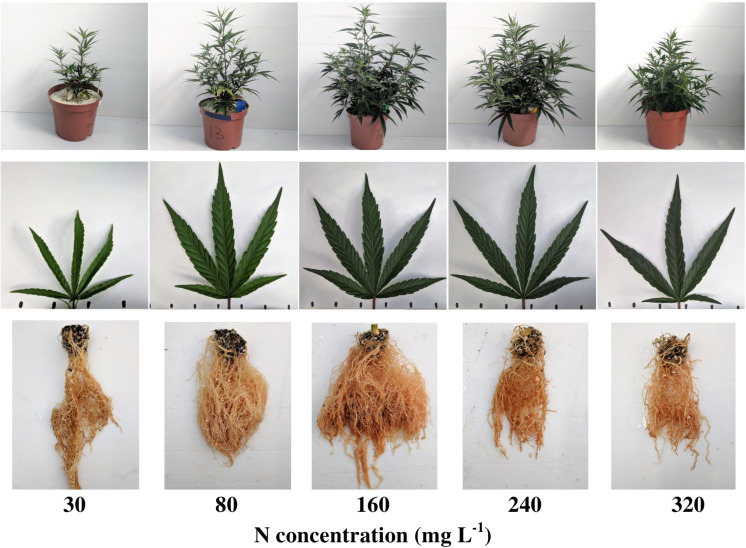
Visual appearance of the plants (top row), leaves (middle row), and roots (bottom row) under increasing N supply. From left to right: 30, 80, 160, 240, 320 mg L^−1^ N. Leaf images are of the youngest, fully developed leaf on the main stem, taken 31 days after the initiation of the fertigation treatments.

The visual appearance of the shoot, leaves, and roots reflects the plant response to the level of N supplied (Figure 1). Plants supplied with 30 mgL^−1^ N showed typical visual signs of N deficiency, including restricted growth and development and apparent leaf chlorosis. The 80 mgL^−1^ N treatment demonstrated restricted growth of the shoot and the root, and chlorosis was detected mainly on the lower-older leaves on the main stem. Plant growth, development, and coloration were optimal under 160 and 240 mgL^−1^ N supply, but root development was restricted at 240 mgL^−1^ N. The 320 ppm N treatment demonstrated visual signs of N overdose, i.e., smaller plant and plant organs, and a dark-green color (Hauck et al., 1984).

### Plant Growth and Development

**FIGURE 2:**
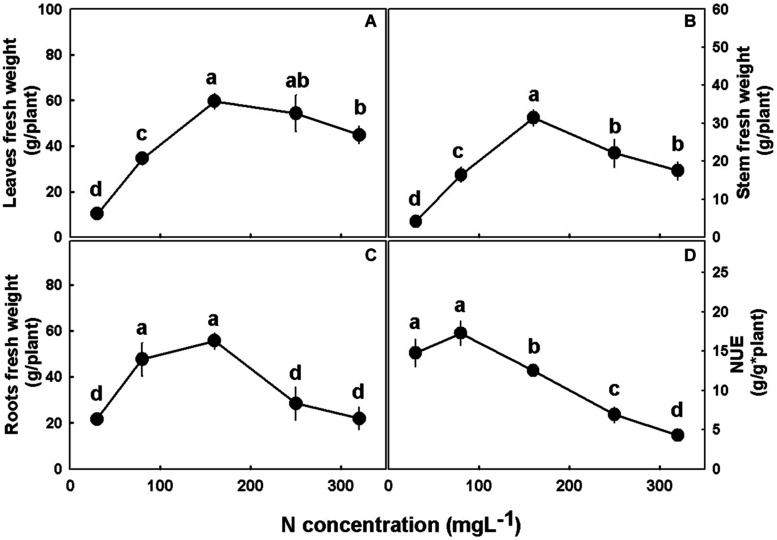
Effect of N nutrition on plant biomass in cannabis plants. Fresh weights of leaves **(A)**, stem **(B)**, and roots **(C)**, and nitrogen use efficiency (NUE) **(D)**. Presented data are averages ± SE (*n* = 5). Different small letters above the means represent significant differences between treatments by Tukey HSD test at α = 0.05.

**FIGURE 3:**
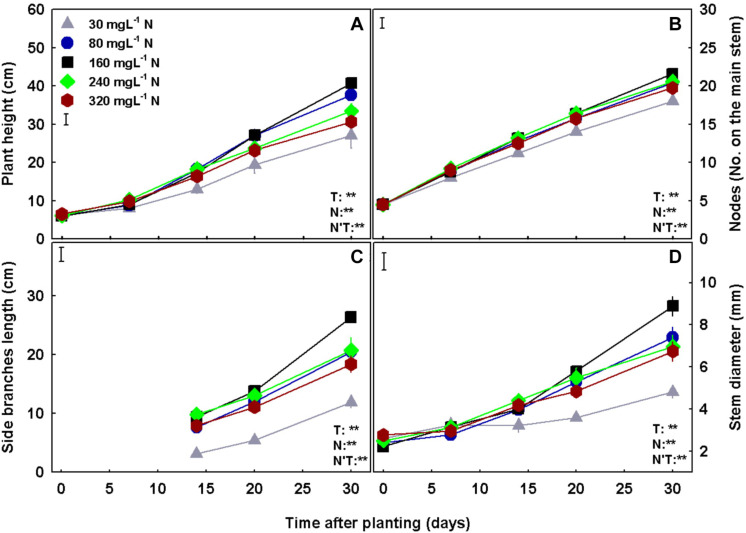
Effect of N concentration on the development of medical cannabis plants at the vegetative growth phase. Plant height **(A)**, number of nodes on the main stem **(B)**, length of side branches **(C)**, and stem diameter **(D)**. Presented data are averages ± SE (*n* = 5). Results of two-way ANOVA indicated as **P < 0.05, F-test; NS, not significant *P* > 0.05, F-test. The bars represent the LSD between means at *P* ≤ 0.05. In the ANOVA results, N’T represents the interaction between N and Time.

Plant growth, development, and morphology were affected by N supply (Figures 2, 3). The low N treatments (30–80 mgL^−1^) were insufficient to support optimal shoot and root development, and cultivation under these concentrations resulted in lower stem radial growth (Figure 3D), side branches elongation (Figure 3C), plant elongation and leaves, stems, and root biomass accumulation (Figures 2A–C) as compared to higher N supply. The plants demonstrated sensitivity to high N supply, and supply above 160 mgL^−1^, was found to be excessive, restricting growth and biomass deposition. Optimal plant growth and development were obtained under 160 mgL^−1^ N supply. Deficiency, optimal, and excessive concentrations were similar for shoot and root biomass accumulation (Figure 2). NUE was also affected by N supply, revealing a high efficiency in the 30–80 mgL^−1^ N treatments, and a decline in efficiency with further increase in N supply (Figure 2D).

### Macronutrient Concentration

**FIGURE 4:**
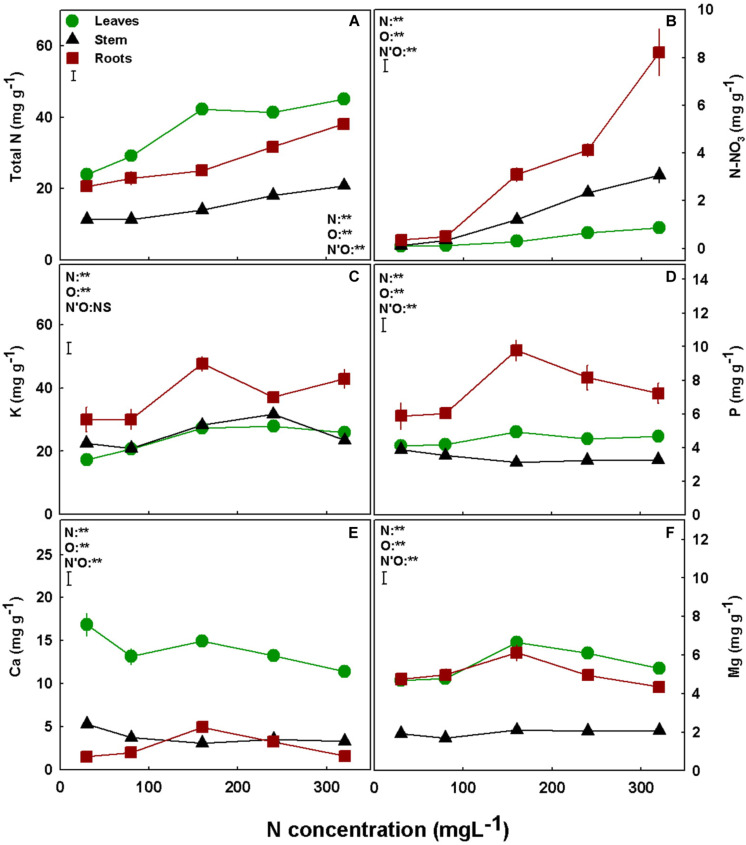
Effect of N supply on macronutrient concentrations in leaves, stem and roots of medical cannabis plants. Total N **(A)**, N-NO_3_ **(B)**, K **(C)**, P **(D)**, Ca **(E)**, and Mg **(F)**. Presented data are averages ± SE (*n* = 5). Results of two-way ANOVA indicated as **P < 0.05, F-test; NS, not significant *P* > 0.05, F-test. The bars represent the LSD between means at *P* ≤ 0.05. In the ANOVA results, N’O represents the interaction between N and plant organ. Where not seen, SEs are smaller than the symbol size.

Nitrogen is well known for its impact on plant uptake and *in-planta* transport. N supply affected N uptake into the roots and translocation to the shoot, as total N contents of the roots, leaves, and stem generally increased with the increase in N supply throughout the concentration range tested (Figure 4A). The same response was apparent also for N-NO_3_ accumulation (Figure 4B). P accumulation in the roots demonstrated optimum curvature, with an optimum at 160 mgL^−1^ N, while the accumulation in the shoot organs was not significantly influenced by N supply (Figure 4D). K uptake and accumulation was lowest under low N supply, in the range of 30–80 mgL^−1^ N in all plant organs (Figure 4C). Under higher N supply of 160–320 mgL^−1^ N, the accumulation was organ specific: K concentration in the roots was significantly lower under 240 mgL^−1^ N compared to 160 mgL^−1^ N; in the stem it was significantly lower at 320 mgL^−1^ N, and in the leaves there was no significant difference in K concentration between treatments (Figure 4C). The two double-positive charged macronutrients, Ca and Mg, demonstrated a similar response to N supply (Figures 4E,F). For both minerals, the concentration in the stem was low and was not significantly influenced by N supply, while root concentrations showed bell-shape response curves with a peak at 160 mgL^−1^ N. In the leaves, Mg showed a similar trend to the one found in the roots, while for Ca the highest accumulation was found under 30 mgL^−1^ N (Figures 4E,F).

### Micronutrients and Na

**FIGURE 5:**
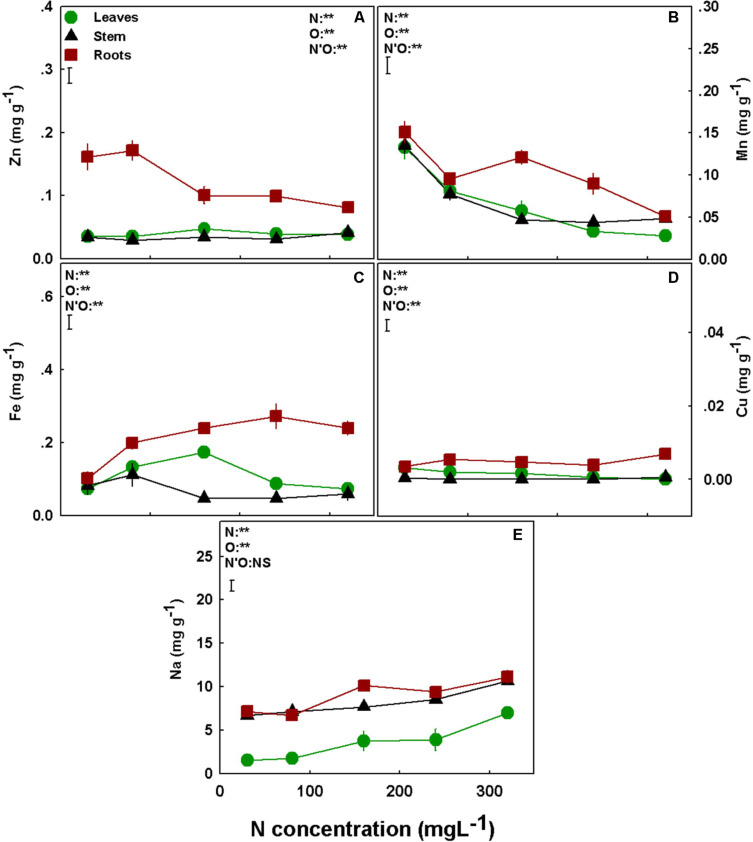
Effect of N supply on micronutrients and Na concentrations in leaves, stem, and roots of medical cannabis plants. Zn **(A)**, Mn **(B)**, Fe **(C)**, Cu **(D)**, and Na **(E)**. Presented data are averages ± SE (*n* = 5). Results of two-way ANOVA indicated as **P < 0.05, F-test; NS, not significant *P* > 0.05, F-test. The bars represent the LSD between means at *P* ≤ 0.05. In the ANOVA results, N’O represents the interaction between N and plant organ. Where not seen, SEs are smaller than the symbol size.

The accumulation of the micronutrients in the plant was significantly affected by the level of N supplied. Interestingly, most micronutrients tested (Zn, Mn, Fe, Cu) demonstrated a clear trend of preferential accumulation in the root, with lower transport to- and accumulation in shoot organs (Figures 5A–D). A second apparent common trend is a lack of influence of N supply on the accumulation of micronutrients in the stem. With the exception of Mn, that was significantly higher in stems compared to other plant organs in the N supply range of 30–80 mgL^−1^, the levels of all other micronutrients tested were very low in the stem and not significantly affected by the N treatments (Figures 5A–D). Apart from these common trends, the micronutrients demonstrated specific accumulation responses. Specifically, Zn concentration in the roots was highest under low N supply (up to 80 mgL^−1^ N), and lower and steady under 160–320 mgL^−1^ N, while the concentrations in the leaves and stems were lower and unaffected by the N treatments (Figure 5A). Under high N supply (160–320 mgL^−1^ N) Mn concentrations in the roots were higher than in the shoot, and highest at the 160 mgL^−1^ N treatments; however, Mn concentration in the leaves and stem declined throughout the N range with the increase in N supply (Figure 5B). Fe concentration in the leaves and the stems showed an optimum curve response (with a peak at 160 mgL^−1^ N and 80 for the leaves and stems, respectively), while in the roots further increase after 160 mgL^−1^ N did not affect Fe concentration significantly (Figure 5C). Cu concentration in the plant organs was lower than all other studied nutrients, and was not significantly affected by the N treatments (Figure 5D). Unlike the micronutrients, Na concentration of all plant organs increased with the increase in N supply but overall the concentrations were low (<11 mg g^−1^) (Figure 5E).

### Gas Exchange and Photosynthesis

**FIGURE 6:**
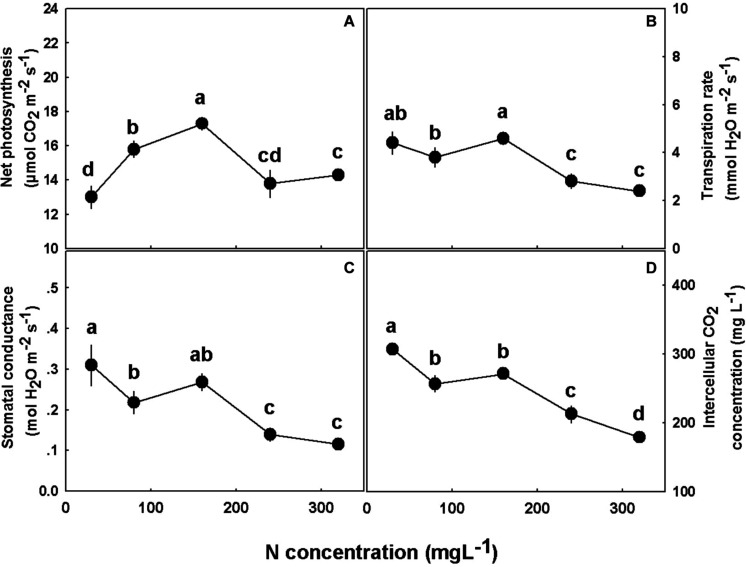
Effect of N concentration on gas exchange in cannabis leaves. Net photosynthesis rate **(A)**, transpiration rate **(B)**, stomatal conductance **(C)**, and intercellular CO_2_ concentration **(D)**. Presented data are averages ± SE (*n* = 5). Different small letters above the means represent significant differences between treatments by Tukey HSD test at α = 0.05.

N supply affected the physiological and gas exchange parameters studied. Photosynthesis rate demonstrated an optimum response to elevation of N supply, with the highest rate at 160 mgL^−1^ N (Figure 6A). Transpiration rate, stomatal conductance, and intercellular CO_2_ concentration displayed a similar response pattern, which was steady under 30–160 mgL^−1^ N range, and decrease with further increase in N supply (Figures 6B–D).

### Water Relations and Photosynthetic Pigments

**FIGURE 7:**
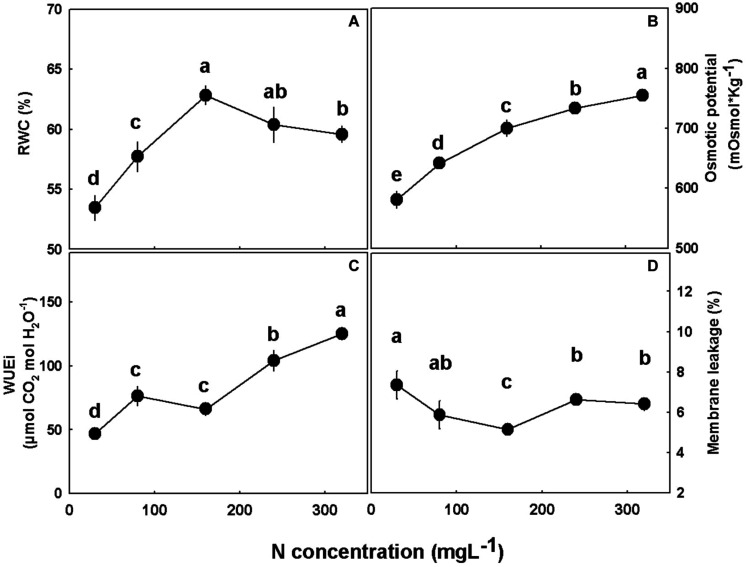
Physiological characteristics of medical cannabis plants. Relative water content (RWC) **(A)**, osmotic potential **(B)**, intrinsic water use efficiency (WUEi) **(C)**, and membrane leakage **(D)**. Presented data are averages ± SE (*n* = 5). Different small letters above the means represent significant differences between treatments by Tukey HSD test at α = 0.05.

Water relations of the plant tissues were considerably affected by the N treatments (Figure 7). RWC increased (by 15%) with the increase in N supply from 30 to 160 mgL^−1^, but with further elevation of N supply from 160 to 320 mgL^−1^ it decreased moderately (by 5%) (Figure 7A). Leaf osmotic potential, as well as the intrinsic water use efficiency (WUEi), increased with the increase in N supply throughout the N concentration tested (Figures 7B,C). Membrane leakage was lower under 160 mgL^−1^ N compared to all other N levels tested (Figure 7D).

**FIGURE 8:**
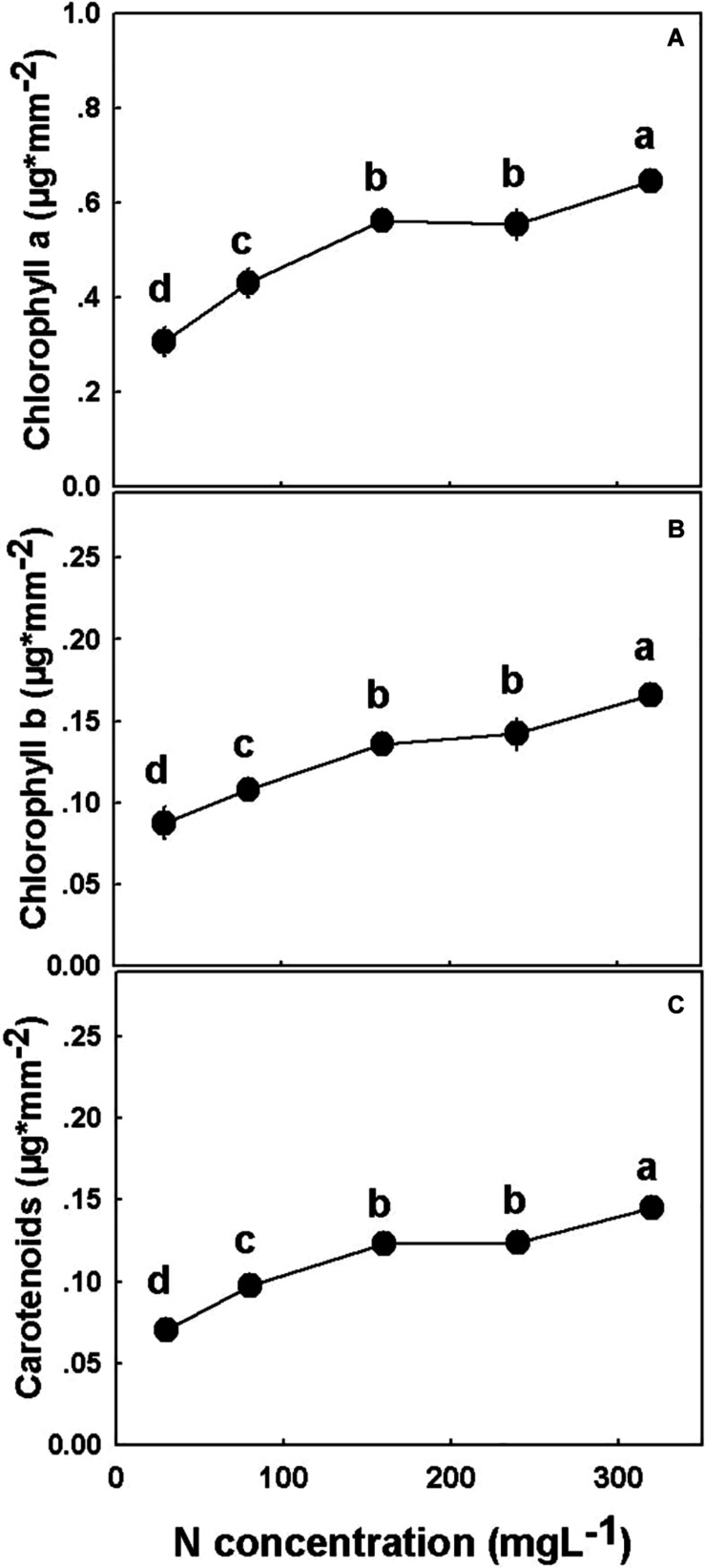
Effect of N application on the concentration of photosynthetic pigments in cannabis. Chlorophyll *a* **(A)**, chlorophyll *b* **(B)**, and carotenoids **(C)**. Presented data are averages ± SE (*n* = 5). Different small letters above the means represent significant differences between treatments by Tukey HSD test at α = 0.05. Where not seen, SEs are smaller than the symbol size.

The concentrations of all three photosynthetic pigments tested, chlorophyll *a*, chlorophyll *b*, and carotenoids, increased significantly with the elevation of N supply from 30 to 160 mgL^−1^ N, and from 240 to 320 mgL^−1^, but was unchanged under 160–240 mgL^−1^ N (Figure 8).

### Irrigation and Leachate Solutions

**FIGURE 9:**
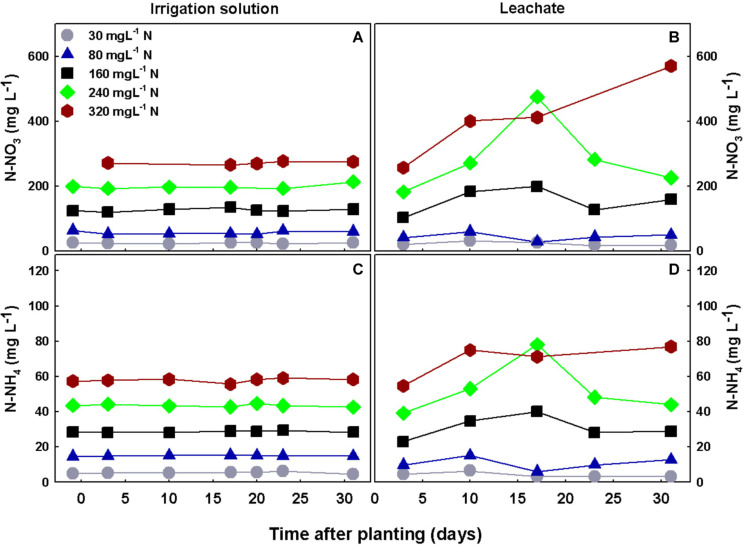
Concentrations of N-NO_3_ and N-NH_4_ in the irrigation solutions **(A,C)**, and leachates **(B,D)**, throughout the experiment duration.

The concentrations of nutrients in the irrigation solutions were steady throughout the experiment and were in-line with the target concentrations, showing proper control of the treatment conditions (results for N-NO_3_ and N-NH_4_ are presented in Figures 9A,C). Differences between nutrient concentrations in the irrigation solution and the leachate can point at the plant requirement and uptake. N-NO_3_ and N-NH_4_ concentrations in the leachates of the low N treatments (30–80 mgL^−1^ N supply) did not differ significantly and were generally lower than the concentrations in the irrigation solutions, demonstrating that N was taken up at rates higher than water. Under higher levels of N supply, the concentrations in the leachates were usually higher than the concentration in the irrigation water demonstrating excess supply. Specifically under 160 mgL^−1^ N-NO_3_ and N-NH_4_ in the leachates increased moderately over time and were generally higher than in the irrigation solution, by 22–48 and 8–38%, respectively, throughout the experiment duration. Under higher N supply of 240 mgL^−1^, N-NO_3_ and N-NH_4_ concentrations in the leachate were higher by 37–141 and 22–83%, respectively, than in the irrigation solution. While under 320 mgL^−1^ N supply, N-NO_3_ concentration in the leachate was almost twice the concentration in the irrigation solution throughout the experiment and N-NH_4_ was also higher than in the irrigation solution (Figures 9B,D). This demonstrates that although plant N uptake increased throughout the N range tested (Figures 4A,B), under supply levels higher than 160 mgL^−1^ N the plant received more N that it can consume. pH levels in the irrigation solutions were between 5.7 and 5.9 and did not differ between treatments, while EC levels increased with N supply and were 1615, 1730, 2080, 2510, and 2760 μs/cm, for the 30, 80, 160, 240, and 320 mgL^−1^ N treatments, respectively.

## Discussion

Nitrogen supply is one of the most common limiting factors for plant growth and development (Weisler et al., 2001; Das and J, 2015; Bouchet et al., 2016; Yadav et al., 2017), due to its essential role as a substrate for the biosynthesis of many vital cell components such as nucleic acids, amino acids, the photosynthetic pigment chlorophyll, and many more (Haynes, 1986; Budil and Thurnauer, 1991; Hawkesford et al., 2012; Wen et al., 2019; Zhang et al., 2019). Insufficient availability of N to plant cells results in shortage of these key metabolites, restricted cell metabolism, and thereby a reduction in most plant processes and developmental delays. It is therefore not surprising that N supply considerably affected the physiological and morphological status of the cannabis plants tested.

Nitrogen supply positively correlated with concentrations of photosynthetic pigments in the leaves (Figure 8), and with net photosynthesis rates (Figure 6A) up to 160 mgL^−1^ N supply. N is a major constituent in these pigments, i.e., each chlorophyll molecule contains four atoms of N (Budil and Thurnauer, 1991). As was demonstrated before for numerous plant species (Evans, 1989; Dordas and Sioulas, 2008; Najm et al., 2012), in cannabis as well, the increase in N supply supported enhanced pigment production. Since one of the major factors influencing the rate of photosynthesis is the concentration of photosynthetic pigments (Emerson, 1929; Frank et al., 1991; Maekawa and Kokubun, 2005; Ionescu et al., 2009), we suggest that the low rate of photosynthesis under 30–80 mgL^−1^ N reflects the low concentration of these pigments (Figure 6A).

Moreover, N supply is known to affect proteins of the Calvin cycle and thylakoids, which represent the majority of leaf N (Evans, 1989), forming a link between leaf N concentration and leaf CO_2_ assimilation (Sinclair and Horie, 1989; Sung et al., 2013). The relation between leaf N, chlorophyll concentration, and photosynthesis is apparent in our results for low N supply—up to 160 mgL^−1^ N. Further increase in N supply and concentrations of photosynthetic pigments at the 240–320 mgL^−1^ N range were not accompanied by further increase in photosynthesis, since at those levels the photosynthetic pigments were no longer the limiting factor. Rather, the decline in photosynthesis under high N supply resulted from stomatal closure, which was accompanied by further reduction in stomatal conductance, transpiration rate, and intercellular CO_2_ (Figure 6).

Another parameter affected by concentrations of the photosynthetic pigments is leaf color (Figure 1). The chlorotic-pale green-yellowish leaves of the low N treatments reflect the low levels of chlorophyll, while the dark green leaves at the 160–320 mgL^−1^ N range indicate a sufficient level of pigments. Moreover, the similar leaf color at the 160–320 mgL^−1^ N range strengthens the conclusion that at this range the pigments are no longer the limiting factor for carbon fixation, as already under 160 mgL^−1^ N the impact of the pigments and the rate of photosynthesis are at their maximum (Figure 6A).

Photosynthesis is the main process governing plant production and growth, as photosynthesis rate indicates on carbohydrate production, and consequently biomass production and accumulation (Pearcy et al., 1981; Whitmarsh and Govindjee, 1999; Malhi et al., 2015). The response of the cannabis plant growth (e.g., plant height, stem width and side branches length; Figure 3) and biomass accumulation into the plant organs (Figures 2A–C) to N supply reflects the photosynthetic rate of the plant (Figure 6A), emphasizing the interplay between plant metabolism, energy status, and plant growth. The effect of N supply on plant development was visibly apparent (Figure 1) with optimal leaf, shoot, and root development identified under 160 mgL^−1^ N, and impaired growth under higher and lower N supply. Unlike other growth parameters, node formation was not affected by the wide range of N supply tested in the present study (Figure 3B), demonstrating insensitivity to N availability. In other plants, node formation was indeed demonstrated to be regulated by other factors, primarily temperature, rather than by N supply (Ingram and Hilton, 1986; Berghage, 1998; Gabryszewska, 2011; Peart and Shoup, 2018).

As a prime macro-mineral that is utilized in large quantities by all plant organs, N is known for its impact on uptake and transport of other plant mineral nutrients by the roots (Miller et al., 1976; Hegde, 1987; Cimrin et al., 2004; Shivay et al., 2007; White, 2012; Savvas et al., 2018), which may lead to changes in ion transport and translocation in the plant. Although total N and N-NO_3_ accumulation in the roots and stem increased with N supply throughout the N range tested, in the leaves, only N-NO_3_ concentration increased with N supply while the total N was not affected by N levels greater than 160 mgL^−1^ (Figures 4A,B). Total N is composed mainly of N-containing organic metabolites which compose the largest N reservoir in the leaf, as demonstrated before for other plant species (Evans, 1989; Sinclair and Horie, 1989), and N-NO_3_ is only a small fraction of this reservoir. The results therefore teach us that under N supply > 160 mgL^−1^ there was a decline in production and accumulation of N-containing organic metabolites in the leaves (Figures 4A,B). This decline in N-containing organic metabolites is in accord with the plant overall toxicity response to oversupply of N, as will be discussed later.

The optimum-shape response curves to increased N inputs that were obtained for the majority of macronutrients (P, K, Ca, Mg; Figure 4) is quite different from the response we reported before for K supply (Saloner et al., 2019), and can be correlated to the plant vitality status, i.e., high nutrient uptake and accumulation which are energy-consuming processes, reflect prime functional morpho-physiological status (White, 2012). Another worth-noting trend is that lower N supply promoted accumulation of metals such Mn, Ca, and Zn in the plant (Figures 4E, 5A,B), while Fe declined under the lower N supply (Figure 5C), demonstrating specificity in the response of ion uptake mechanisms to N supply. Enhanced supply of specific ions to the root zone can affect uptake and accumulation of other nutrients in the plant by competition for uptake. NH_4_ is well known to compete with other cations such as Mg, Mn, and Ca (Geraldson, 1957; Cox and Reisenauer, 1977; Rayar and van Hai, 1977; Van Beusichem et al., 1988; White, 2012), and this was apparent also in the present study as the levels of these minerals reduced with the increase of N supply over 160 mgL^−1^ (Figures 4E,F, 5B).

The osmotic potential response to N-input reflects trends of nutrient accumulation in the leaves (Figure 7B). Since N and N-NO_3_ concentrations were greatly elevated with the increase in N supply, while concentrations of most other macronutrients were not highly affected, the increase in osmotic potential with N and N-NO_3_ supply correlates with their accumulation in the tissue sap. The difference in trends between the osmotic potential curve that increases throughout the N concentration gradient, and the RWC curve (Figure 7A), which displays a maximum curvature, with drier leaves in both extreme N treatments, supports that the increase in osmotic potential is due to specific accumulation and not solely a result of tissue drying. This notion is supported by results of bean to N supply (Guo et al., 2007).

In agricultural systems, the plant is considered a production unit, and in this respect, it is vital to examine its efficiency. Since elevated N supply promoted a better functioning photosynthetic system, due to the high content of photosynthetic pigments, stomatal conductance could be reduced (Figure 6C). This decline diminished water loss by transpiration (Figure 6B), but at concentrations higher than 160 mg L^−1^ also decreased the intercellular CO_2_ concentration (Figure 6D). The significant reduction in transpiration and hence water loss (Figure 6B) made the plant more efficient in terms of water use (Figure 7C). Despite the considerable increase in water use efficiency, N use efficiency declined as N supply increased (Figure 2D), meaning that despite the higher biomass production under the 160 mgL^−1^ N level, the plants produced less biomass per N unit. Hence, the plants are less efficient in terms of N use at the higher N levels. As nitrate was elevated in the roots with the increase in N supply (Figure 4B), it is presumed that the decrease in plant N use efficiency is due to a restricted nitrate reduction in the plant roots. Restricted ability to reduce nitrate in the roots may result from a feedback inhibition of nitrate reductase from the NH_4_^+^ supplied and from ammonia-derived metabolites, as was demonstrated for many plants before (Srivastava, 1980; Konnerup and Brix, 2010; Piwpuan et al., 2013), or from a inactivity of nitrate reductase in roots, and this issue should be further investigated.

The decline in metabolism and phenotypic development under high N supply (240 mgL^−1^ N and above) in the cannabis plant is the outcome of two main damage avenues; namely, specific detrimental effects of the nitrate or ammonium ions, and non-specific negative effects of high concentrations. Nitrate and ammonium, like any nutrient ions, have optimal concentration range in plant tissues in terms of plant response to N dose (Broadley et al., 2012; Hawkesford et al., 2012). When nitrate level exceeds the threshold of tolerable level (i.e., the toxicity threshold), it can induce cell damage by various mechanisms. First, nitrate assimilation elevates intracellular pH, because nitrate reduction to nitrite is a proton-consuming process that enriches the cytosol with OH^–^ (Britto and Kronzucker, 2005). This elicits enhanced production of organic acid anions to stabilize cell pH and maintain the anion–cation biochemical balance, but has a downside as well since the organic acid biosynthesis is energy-consuming and may dwindle energy availability in the cells thus contributing to growth restriction (Hauck et al., 1984). Furthermore, excess nitrate accumulation can be toxic as it results in nitrite and nitric oxide production—two molecules which are highly toxic to plants when over-accumulate (Hauck et al., 1984; Lamattina et al., 2003; Anjana and Iqbal, 2007; Zheng et al., 2016). As NH_4_ supply was elevated as well at the high N treatments, reaching up to 58 mgL^−1^ N-NH_4_, it cannot be excluded that the detrimental effects were caused by NH_4_ overdose, a well known and discussed phenomenon in plant nutrition (Hauck et al., 1984; Bernstein et al., 2005; Hawkesford et al., 2012). *C. sativa* responses to NO_3_/NH_4_ ratio, and the involved mechanisms are yet unknown and are currently under investigation in our laboratory. A non-specific effect of N toxicity may involve a non-specific concentration-driven osmotic stress in the plant tissues, i.e., a salinity effect. Such an effect is apparent from the rise in osmotic potential of the leaf tissue sap (with the increase in N supply and N-NO_3_ concentration in the leaves; Figures 4B, 7B). The results also point at involvement of oxidative damage under N access and deficient conditions. Various abiotic stress conditions, including mineral deficiencies and toxicities, and N nutrition in particular, are accompanied by oxidative stress, i.e., unregulated increase in concentration of reactive oxygen species (ROS) in the plant tissue (Kong et al., 2013; Chokshi et al., 2017; Choudhury et al., 2017; Bellegarde et al., 2019). N deficiency and toxicity levels correlated in the present study with reduced membrane integrity, e.g., increased membrane leakage, that reflects oxidative damage and especially lipid peroxidation (Liu and Huang, 2000; Bernstein et al., 2010; Kravchik and Bernstein, 2013; Teissie, 2017) suggesting a role of oxidative stress in the deficiency and toxicity responses.

Nitrogen overdose often causes excessive production of organic N molecules as was discussed above, consuming high amounts of metabolic energy, which reduces energy and carbohydrates availability, and delays biomass production. Furthermore, N uptake and assimilation are energy-consuming processes which increase under high N supply (Mengel and Viro, 1978; Zapata et al., 1987; Huppe and Turpin, 1994; Hawkesford et al., 2012) and further dwindle energy availability. Ion uptake, that is an energy consuming process, indeed correlated in the present study with carbon fixation, and a decline in uptake and accumulation of vital nutrients P, Ca, Mg, and Mn took place at the high N treatments (Figures 4D–F, 5B).

## Conclusion

In summary, we found growth retardation under low N supply (30–80 mgL^−1^) to result from restricted photosynthetic pigments biosynthesis, carbon fixation, and impaired water relations. Excess uptake of N under supply larger than the optimal level of 160 mgL^−1^ N induced growth retardation by ion-specific toxicity or an indirect induced restriction of carbon fixation. We conclude that the morpho-physiological function under long photoperiod is optimal at 160 mgL^−1^ N, in the medical cannabis cultivar tested.

## Data Availability Statement

All datasets presented in this study are included in the article/supplementary material.

## Author Contributions

NB planned the experiments. AS carried out the experiments. NB and AS wrote the manuscript. Both authors contributed to the article and approved the submitted version.

## Conflict of Interest

The authors declare that the research was conducted in the absence of any commercial or financial relationships that could be construed as a potential conflict of interest.
